# Automatic planning of the lower extremities for total marrow irradiation using volumetric modulated arc therapy

**DOI:** 10.1007/s00066-022-02014-0

**Published:** 2022-11-03

**Authors:** Nicola Lambri, Damiano Dei, Victor Hernandez, Isabella Castiglioni, Elena Clerici, Leonardo Crespi, Chiara De Philippis, Daniele Loiacono, Pierina Navarria, Giacomo Reggiori, Roberto Rusconi, Stefano Tomatis, Stefania Bramanti, Marta Scorsetti, Pietro Mancosu

**Affiliations:** 1 Department of Biomedical Sciences, Humanitas University, via Rita Levi Montalcini 4, 20072 Pieve Emanuele, Milan, Italy; 2 Radiotherapy and Radiosurgery Department, IRCCS Humanitas Research Hospital, via Manzoni 56, 20089 Rozzano, Milan, Italy; 3 Department of Medical Physics, Hospital Universitari Sant Joan de Reus, IISPV, Tarragona, Spain; 4 Department of Physics “G. Occhialini”, University of Milan-Bicocca, piazza della Scienza 2, 20126 Milano, Italy; 5 Dipartimento di Elettronica, Informazione e Bioingegneria, Politecnico di Milano, Milan, Italy; 6 Human Techopole, Centre for Health Data Science, Milan, Italy; 7 Bone Marrow Transplantation Unit, IRCCS Humanitas Research Hospital, Milan, Rozzano, Italy

**Keywords:** Radiotherapy, Automation, TMI, VMAT, Field junction

## Abstract

**Purpose:**

Total marrow (and lymphoid) irradiation (TMI-TMLI) is limited by the couch travel range of modern linacs, which forces the treatment delivery to be split into two plans with opposite orientations: a head-first supine upper-body plan, and a feet-first supine lower extremities plan. A specific field junction is thus needed to obtain adequate target coverage in the overlap region of the two plans. In this study, an automatic procedure was developed for field junction creation and lower extremities plan optimization.

**Methods:**

Ten patients treated with TMI-TMLI at our institution were selected retrospectively. The planning of the lower extremities was performed automatically. Target volume parameters (CTV_J‑V_98%_ > 98%) at the junction region and several dose statistics (D_98%_, D_mean_, and D_2%_) were compared between automatic and manual plans. The modulation complexity score (MCS) was used to assess plan complexity.

**Results:**

The automatic procedure required 60–90 min, depending on the case. All automatic plans achieved clinically acceptable dosimetric results (CTV_J‑V_98%_ > 98%), with significant differences found at the junction region, where D_mean_ and D_2%_ increased on average by 2.4% (*p* < 0.03) and 3.0% (*p* < 0.02), respectively. Similar plan complexity was observed (median MCS = 0.12). Since March 2022, the automatic procedure has been introduced in our clinic, reducing the TMI-TMLI simulation-to-delivery schedule by 2 days.

**Conclusion:**

The developed procedure allowed treatment planning of TMI-TMLI to be streamlined, increasing efficiency and standardization, preventing human errors, while maintaining the dosimetric plan quality and complexity of manual plans. Automated strategies can simplify the future adoption and clinical implementation of TMI-TMLI treatments in new centers.

**Supplementary Information:**

The online version of this article (10.1007/s00066-022-02014-0) contains supplementary material, which is available to authorized users.

## Introduction

Total body irradiation (TBI) is a radiotherapy (RT) technique adopted in conditioning regimens for patients undergoing hematopoietic cell transplantation in multiple myeloma, acute leukemia, and lymphomas [1]. Standard TBI techniques involve irradiation of the whole body and are therefore unable to cover the target volume without exposing healthy tissues to the full planned dose. Late toxicities induced by TBI could potentially be avoided by adoption of more targeted forms of RT, such as total marrow (and lymphoid) irradiation (TMI-TMLI) [2]. The aim of TMI-TMLI is to optimize coverage of the hematopoietic target and lymphoid tissues while sparing radiation to the organs at risk (OARs).

Dosimetric studies have demonstrated the technical feasibility of TMI-TMLI delivered using helical tomotherapy (HT) [3–5] as well as C‑arm linear accelerators with intensity-modulated radiation therapy with large static fields (sf-IMRT) [6–8], and, more recently, using volumetric modulated arc therapy (VMAT) [9–11]. For all these approaches, the authors obtained adequate target coverage and dose reductions in OARs compared to conventional TBI. At our institute, since October 2010, TMI-TMLI has been delivered using the VMAT technique [12–15].

The dosimetric advantages of TMI-TMLI have resulted in proliferation of many phase I–II clinical trials that have been or currently are under investigation with the aim of improving disease control [2]. Promising clinical data, however, has not led to widespread introduction of TMI-TMLI to replace TBI because of several challenges that need to be addressed. Schultheiss et al. [4] reported manual contouring times of 12–16 h for targets and normal tissues. TMI-TMLI plan optimization is an iterative trial-and-error process that could require several days to obtain adequate dose distributions. Furthermore, the time required to treat a patient with VMAT-based TMI-TMLI could exceed the time required for traditional TBI. Thus, an ad hoc immobilization system should be considered to minimize unwanted patient set-up motion due to the prolonged door-to-door time [12]. All these technical difficulties and the need for a dedicated team still represent a barrier to the adoption of TMI-TMLI [2].

Automatic approaches are sought and could be highly beneficial for the progress of TMI-TMLI. As the number of patients undergoing hematopoietic cell transplantation and who are candidate for irradiation of the whole body is expected to increase in the coming years [16–20], standardization and automation of VMAT-based TMI-TMLI is needed to streamline the planning process of this complicated treatment and to assist centers which will introduce it in the near future. Furthermore, as the majority of modern linacs can deliver VMAT treatments, most centers worldwide could potentially deliver TMI-TMLI using this technique [2].

Most of the TMI-TMLI studies in the literature have focused on the upper part of the body (i.e., above the femurs), where all the OARs are present. However, because of limitations in the couch travel range of both C‑arm and HT linacs (130–150 cm), TMI-TMLI delivery must be split into two parts: one for the upper part of the body (in head-first supine position) and one for the lower extremities (in feet-first supine position). A specific field junction is thus needed to create two mirroring sigmoid dose profiles in the most caudal region of the upper body and most cranial region of the lower extremities, to create a dose distribution that provides both acceptable target coverage and is robust to setup errors in the overlap region.

Recently, some authors described a manual procedure to create a field junction to deliver TMI-TMLI or TBI to the whole body with HT [21–24], while the feasibility of a manually created field junction for VMAT-based TMI-TMLI was investigated at our institute, demonstrating an optimal target coverage and dosimetric junction robustness for patient shifts of up to 10 mm [15]. However, as the whole process is time consuming and error prone, automating the creation of field junctions and optimization of the lower extremities plan could speed up the planning process of TMI-TMLI and reduce potential errors without affecting the resulting dosimetric quality.

In this study, we designed an automatic procedure to optimize the TMI-TMLI lower extremities plan with a robust field junction. We validated the results by comparing several dose statistics and plan complexity with those from the corresponding clinical plans optimized manually. The aim of the present study was to implement this automatic approach and show how it can help to streamline and standardize the planning process of TMI-TMLI treatments.

## Materials and methods

### Simulation and target volume definition

Since 2010, 108 adult patients have been treated at our institute with TMI-TMLI by means of VMAT following an internal protocol approved by the institution’s internal scientific committee (ONC/OSS-04/2013) [25]. According to the protocol, all patients were simulated in supine position and positioned using a home-made dedicated immobilization frame [12, 14]. To cover the total cranial–caudal (CC) extension of a patient, two CT images per patient were reconstructed with a 5-mm slice thickness, one in head-first supine (upper body CT) and a second one in feet-first supine (lower extremities CT). The upper body CT scan extended from the top of the skull to the knees and was acquired in free-breathing mode. Arms were immobilized alongside the body to ensure patient comfort and reproducibility. The lower extremities CT scan extended from the feet to the femoral heads. Between the two acquisitions, the patient was taken off the couch, the immobilization frame was rotated to the feet-first position, and the patient was placed back on the frame.

The planning target volume (PTV) was defined as the individual bones, with the exclusion of hands, mandible, and maxillary structures, to provide an additional margin around the bone marrow. The whole chest wall was considered as part of the PTV to include the breathing motion of the ribs, and the bones of arms and legs were isotropically expanded by 10 mm to account for setup uncertainties and potential intra-fractional motion. For TMLI treatments, the spleen and lymph nodes plus an additional isotropic margin of 5 mm were included into the PTV. Such expansions are based on the results of our previous studies on the reproducibility of the patient positioning in multi-isocenter plans [12, 14].

### Manual planning

All plans were optimized for a Varian TrueBeam equipped with a Millennium multileaf collimator with 40-cm coverage and 14-cm leaf travel per bank. Plan optimizations were performed with the Eclipse (Varian Medical Systems, Palo Alto, CA, USA) treatment planning system (TPS) using the Photon Optimizer (PO, v15) optimization algorithm, while the dose distributions were computed with the analytical anisotropic algorithm (AAA, v15), with a calculation grid resolution of 2.5 mm. The total dose prescription for the patients selected in this study, all TMLI patients, was 2 Gy delivered in a single fraction. All plans were normalized so that 98% of the PTV received 98% of the prescribed dose (PTV‑V_98%_ = 98%).

In our previous study, we described the manual procedure followed to optimize the TMI-TMLI field junction in the overlapping region between the upper body and the lower extremities plans of opposite orientations. Hence, only a brief overview is outlined hereafter, and we refer to the original paper for further details [15].

Because of the limitations in the couch travel range of linacs (130–150 cm), the TMI-TMLI delivery was split into two parts. A first plan on the upper body CT (upper body plan) was optimized using five isocenters for a total of 10 full arcs (360°) of 6 MV with asymmetric jaw settings. Each arc overlapped with the adjacent ones for at least 2 cm on each side, such that the differences in delivered dose distributions with respect to planning due to small patient misalignments between isocenters were minimized [14]. During treatment, cone-beam CT images were acquired at each isocenter to correct for potential patient misalignments. The plans were optimized to achieve adequate coverage of the PTV (upper body PTV) and sparing the doses to OARs following the ALARA principle (as low as reasonably achievable). The most caudal slices (4 cm) of the upper body PTV (i.e., lower femurs) were optimized to obtain a dose gradient falling from 100 to 25% of the prescribed dose. This procedure was necessary to avoid potential hotspots once the junction between the upper body plan and the lower extremities plan was created.

A second plan on the lower extremities CT (lower extremities plan) was optimized with four/six full arcs of 6 MV, using two/three isocenters with the collimator angle at 90° or 5°/355°, depending on the patient’s height, with field overlaps of at least 2 cm (see Fig. S1 and Fig. S2 in the Supplementary Material). The lower extremities plan was optimized taking into account the dose given by the upper body plan in order to produce a homogeneous composite dose distribution at the junction after adding the doses of both the upper body and lower extremities plans. To this aim, the 100%, 75%, 50%, and 25% isodoses of the upper body plan were segmented on the upper body CT and propagated to the co-registered lower extremities CT. The isodoses on the lower extremities CT were then used to create the junction substructures receiving 25%, 50%, 75%, and 100% of the prescribed dose based on where the isodose levels terminated.

### Automatic planning

In this study, a plug-in script was developed for the Eclipse TPS to automate and standardize the planning of TMI-TMLI for the lower extremities. The script was written in the C# programming language using the Eclipse Scripting API (ESAPI, v15.6) and can be executed within the Eclipse External Beam Planning module.

**Fig. 1:**
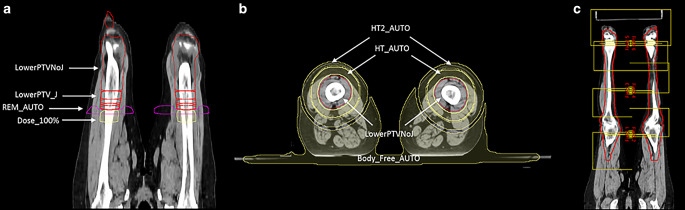
Frontal and transverse views of the main structures generated by the plug-in script on the lower extremities CT (**a**,**b**) and the isocenters and fields placed by the script for plan optimization (**c**). In the text, LowerPTVNoJ and LowerPTV_J are referred to as PTVNoJ and PTV_J, respectively. *LowerPTVNoJ* lower extremities planning target volume (PTV) excluding the junction, *LowerPTV_J* junction structure, *HT_AUTO*, *HT2_AUTO* healthy tissue, *REM_AUTO* remove structure

The script creates a new plan for the lower extremities and generates the junction structures and control structures as shown in Fig. 1a,b, mimicking the manual procedure described in the previous section. Next, the script places three isocenters and six full arcs with a collimator angle at 90°, ensuring 2‑cm overlap between adjacent fields, as shown in Fig. 1c. The isocenters are equally spaced in the CC direction and placed at the center of the lower extremities PTV, with the third isocenter on the feet shifted up 3 cm to better cover the feet under the beam of view of the fields. Finally, the script executes one optimization cycle with intermediate dose calculation, restarting at multi-resolution level 3, and calculates the dose distribution normalized such that 98% of the target volume (PTVNoJ) receives 98% of the prescribed dose.

**Fig. 2:**
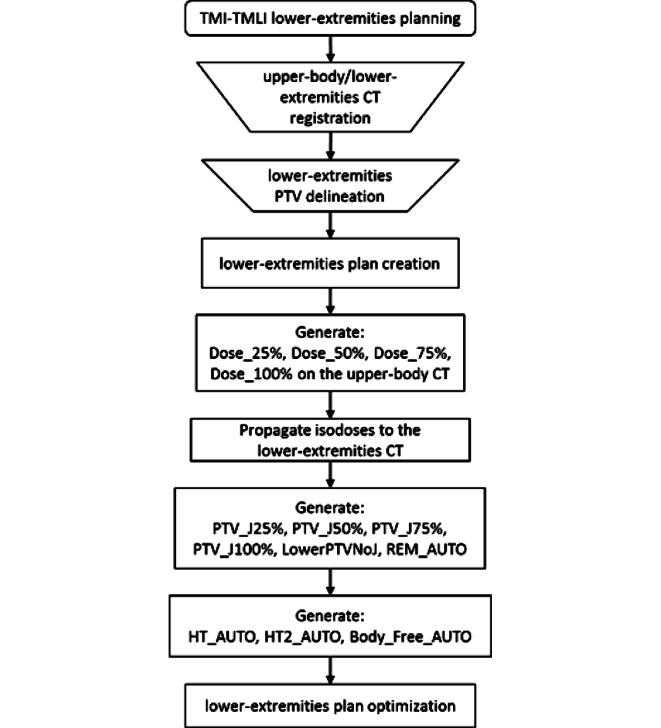
Flowchart describing the workflow of TMI-TMLI lower extremities planning. The steps in rectangular boxes were automated in this study

A detailed description of the script can be found in the Supplementary Material, with a summary of the main structures generated and their characteristics reported in Table S1. The workflow of TMI-TMLI planning for the lower-extremities is reported in the flowchart of Fig. 2, where the steps in rectangles were automated in this study (see Fig. S5 in the Supplementary Material for the complete TMI-TMLI planning flowchart).

### Evaluation

The automatic and manual planning procedures were compared by randomly selecting 10 patients from our clinical database treated with TMI-TMLI (the patients’ demographics are provided in Table S2 of the Supplementary Material). For each patient, a lower extremities structure set containing only the body and lower extremities PTV contours was created, and the lower extremities plan optimization was performed using the procedure described in the sections “Manual planning” and “Automatic planning.” Two plan sums were then created by adding the upper body plan (the same for both lower extremities optimizations) to the manual or automatic lower extremities plan.

An in-house ESAPI script was used to automatically extract target volume parameters and several dose statistics from the plan sums to assess the lower extremities plan optimization outcomes. The two procedures were compared in terms of target coverage of bone and marrow structures within the junction region (CTV_J), with the primary goal CTV_J‑V_98%_ > 98%. The dose received by 98% of the volume (D_98__%_), mean dose (D_mean_), and dose received by 2% of the volume (D_2%_) were evaluated for both CTV_J and the junction structure (PTV_J), while D_mean_ and D_2%_ were considered to assess the dosimetric results on the target volume (PTVNoJ) and healthy tissue (HT_AUTO).

To evaluate potential differences in plan complexity between manual and automatic lower extremities plans, the modulation complexity score (MCS) [26] was computed from the DICOM RT files by means of a software written in MATLAB (Mathworks, Massachusetts, USA) [27]. The MCS combines complexity in segment shapes and beam aperture areas into a single score, and ranges from 0 (maximum complexity) to 1 (no complexity).

### Statistical analysis

Statistical analysis was performed in Python‑3.10.4 with the libraries SciPy‑1.8.1, and pandas‑1.4.2. The Wilcoxon signed-rank test for correlated samples was used to compare the dose objectives between manual and automatic optimizations. A value of *p* < 0.05 was considered statistically significant.

## Results

**Fig. 3:**
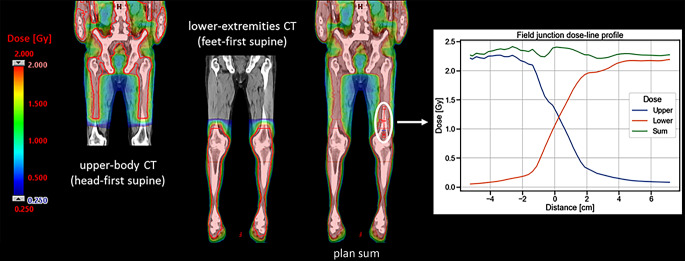
Dose distribution for the upper body plan (manual), lower extremities plan (automatic), and the plan sum for a representative patient. The dose-line profile in the junction region is also reported

The dose distribution of the upper body plan (manual), lower extremities plan (automatic), and the plan sum for a representative patient is shown in Fig. 3. The sigmoid shape of the dose profiles along the CC direction at the junction region proves the feasibility of the automatic procedure. The comparison of the dose line profiles with those of the manual plan can be found in Fig. S6 of the Supplementary Material, where the agreement between the curve demonstrates the robustness of the automatic planning. Analogous results were obtained for all patients.

The total execution time of the plug-in script on a Varian research workstation (Dell Precision 7820, CPU Intel Xeon Silver 4110, RAM 32 GB) was approximately 60–90 min, depending on the complexity of each case.

The main time savings were obtained via the automatic generation of junction and control structures and through the automatic positioning of the isocenters, which took around 3–10 min only. The total time required to perform the same tasks manually ranged from 45 to 120 min, without considering human mistakes that may happen and remain unnoticed until late in the optimization, potentially inducing delays of 1–2 days.

The primary goal on bones and marrow inside the junction structure, i.e., CTV_J‑V_98%_ > 98%, was achieved in all automatic plans. Although this was not the goal of the manual plans, the same criterion was not satisfied by only two of them.

Table 1 reports the plan sum dose objectives for CTV_J, PTV_J, PTVNoJ, and HT_AUTO, as well as the comparison of the MCS between manual and automatic lower extremities plans.

**Table 1:** Median values of the dosimetric results between the plan sums of manual and automatic procedures for the clinical target, junction structure, lower extremities PTV excluding the junction, healthy tissue, as well as the MCS comparison

	CTV_J			PTV_J			PTVNoJ			HT_AUTO		
	D_98%_ (Gy)	D_mean_ (Gy)	D_2%_ (Gy)	D_98%_ (Gy)	D_mean_ (Gy)	D_2%_ (Gy)	D_98%_ (Gy)	D_mean_ (Gy)	D_2%_ (Gy)	D_mean_ (Gy)	D_2%_ (Gy)	MCS
Manual	2.00 (1.82, 2.06)	2.16 (2.02, 2.18)	2.31 (2.22, 2.36)	1.89 (1.63, 1.96)	2.12 (1.96, 2.15)	2.30 (2.21, 2.35)	1.96 –	2.15 (2.05, 2.21)	2.32 (2.16, 2.41)	1.57 (1.48, 1.66)	2.15 (2.07, 2.23)	0.12 (0.10, 0.22)
Automatic	2.05 (2.01, 2.23)	2.22 (2.16, 2.35)	2.39 (2.25, 2.50)	1.92 (1.78, 2.04)	2.17 (2.10, 2.29)	2.37 (2.24, 2.50)	1.96 –	2.13 (2.07, 2.28)	2.29 (2.19, 2.51)	1.59 (1.51, 1.69)	2.11 (2.02, 2.26)	0.12 (0.10, 0.17)
Pairwise %diff	4.2%	3.3%	2.9%	1.6%	2.6%	2.9%	–	−0.2%	−0.4%	2.1%	−0.8%	−8%
*p*-value	< 0.01*	< 0.01*	< 0.01*	> 0.05	< 0.03*	< 0.02*	–	> 0.05	> 0.05	> 0.05	> 0.05	> 0.05

*CTV_J* clinical target, *PTV_J* junction structure, *PTVNoJ* lower extremities planning target volume (PTV) excluding the junction, *HT_AUTO* healthy tissue, *MCS* modulation complexity score, *Pairwise %diff* median of the pairwise percentage differences between automatic and manual procedures, *D*_*x%*_ dose received by x% of the volume.

Numbers in parentheses represent the minimum and maximum values

*Significant *p*-value

Overall, the dose statistics revealed small variations (≤ 4.2%) in the calculated dose between manual and automatic optimizations. Significant differences were found for CTV_J, where the automatic procedure generated plans with slightly increased dose statistics on average. Accordingly, significant differences were found for the junction structure, where the median PTV_J‑D_mean_ increased from 2.12 to 2.17 Gy (median pairwise difference 2.6%, *p* < 0.03) and the median PTV_J‑D_2%_ increased from 2.30 to 2.37 Gy (median pairwise difference 2.9%, *p* < 0.02). Plan normalization was performed on the PTVNoJ structure (i.e., PTV without the junction structure), and a median PTV_J‑D_98%_ of less than 98% of the dose prescription for both manual and automatic optimizations was observed. Furthermore, the differences in dose statistics between manual and automatic optimizations for the target volume PTVNoJ and healthy tissue HT_AUTO were below 2.1%.

**Fig. 4:**
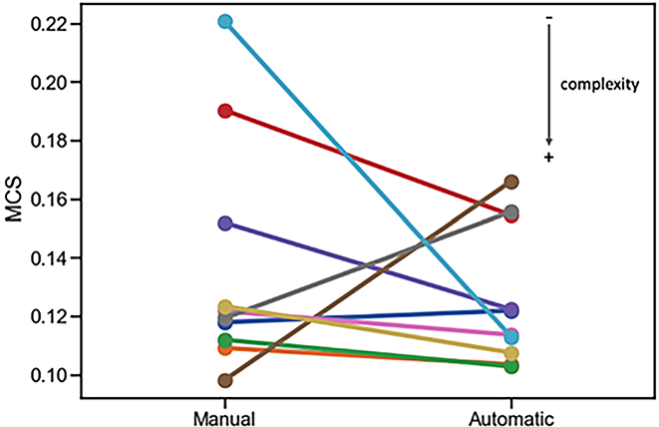
Comparison of the MCS between manual and automatic lower extremities plan pairs

The comparison of the MCS between manual and automatic lower extremities plan pairs is reported in Fig. 4. Overall, the complexity of each automatic plan was similar to that of the corresponding manual plan (range 0.10–0.22). Accordingly, the median MCS remained unchanged (0.12) between manual and automatic optimizations.

Given the small variations observed in the dose statistics and the degree of plan complexity, the manual and automatic plans were considered clinically equivalent. The automation of the lower extremities plan optimization allowed the planning process to be streamlined without affecting the resulting plan quality. Since March 2022, all TMI-TMLI lower extremities plans have been automatically optimized, allowing for a reduction of 2 days in the simulation-to-delivery schedule.

## Discussion

The introduction of automation into the RT treatment planning process is nowadays a central part of clinical practice. Automatic tools offer important advantages, such as reducing the treatment planning time, increasing standardization, and preventing human errors in long and tedious tasks [28].

An example of the advantage of such automation is VMAT-TBI. Currently, there are ongoing efforts toward automation of VMAT-TBI to overcome the limitations of conventional TBI delivered using static open fields. Teruel et al. [29] developed an ESAPI script that produced high-quality VMAT-TBI plans by automating tasks such as isocenter positioning, placement of plan fields, and target and optimization contours creation. Authors reported time savings of 2–3 h, and up to a full day in case of unnoticed manual errors. Simiele et al. [30] developed a binary plug-in script to automate the pre-optimization of VMAT-TBI, and a standalone executable to perform successive optimizations using ESAPI. Planning time was greatly reduced, from 2–3 days to 3–5 h, depending on the complexity of the case. The authors demonstrated that the automatic plans were either superior or equivalent to the corresponding manual plans. Guo et al. [31] developed an in-house MIM (MIM Inc., Cleveland, OH) image processing and segmentation workflow, which reduced the contouring time for VMAT-TBI to 5 min, and a set of in-house planning scripts for the Pinnacle TPS (Philips Inc., Fitchburg, WI) were used by the authors to achieve a planning time of 8–12 h.

All these authors reported that the treatment delivery was split into a VMAT-based plan for the upper body and an anterior-posterior/posterior-anterior (AP-PA) plan for the lower extremities, without directly addressing the quality of the junction matching between the upper and lower plans. The same approach adopted for the lower extremities also appears in the literature of TMI-TMLI studies [5, 6, 10, 11], as the limitation in the couch travel range of both C‑arm and HT linacs forces the irradiation of the whole body to be split into two parts.

The AP-PA approach, placing the patient on a standard couch, has the advantage of simple delivery. However, with AP-PA, multiple junction challenges need to be addressed: (i) the junctions between the fields in the lower extremities plan (as standard linacs allow < 40 cm as maximum field size) and (ii) the dosimetric junction between the upper and lower extremities plans. Since there are no OARs in the lower extremities, a field-in-field approach is not required as for craniospinal irradiation [32]. Treating the lower extremities with VMAT/HT instead of AP-PA allows a more conformal dose distribution and a robust field junction to be obtained at the cost of complicating the plan optimization procedure. Nonetheless, avoiding cold and hotspots inside the lower extremities target could benefit the overall treatment.

Recently, some authors have described a manual procedure to create a field junction to deliver TMI-TMLI or TBI to the whole body with HT [21–24]. However, to the best of our knowledge, an automatic procedure to create a field junction between upper and lower body plans for TMI-TMLI has not yet been proposed. Despite being tested for VMAT-based TMI-TMLI, we argue that the plug-in script presented in this study is fully applicable to both TMI-TMLI and TBI, independently of (i) the delivery technique, such as IMRT or VMAT, and (ii) the dose prescription.

The automatic planning procedure proposed in this study has been successfully introduced in the clinic since March 2022, drastically reducing the time required to optimize a TMI-TMLI lower extremities plan and gaining up to 2 days in time savings. The optimization outcome of the script is evaluated by the planner, who, if necessary, can create additional control structures and slightly tweak the optimization objectives to obtain the desired dose distribution by simply adding one additional optimization cycle.

The script implements a planning procedure designed by experts, and its execution time was compared with the planning time required by an experienced planner. Thus, the script could greatly facilitate the clinical introduction of TMI-TMLI in centers that have limited or no experience in TMI-TMLI treatment planning by reducing the required time and the technical difficulties of the planning optimization, which currently constitute a barrier for the widespread adoption of TMI-TMLI [2]. To this aim, the script executable can be downloaded from the Supplementary Material, and the source code developed in this study is publicly available at https://github.com/nlambriICH/TMIAutomation.

Despite the relatively small patient cohort considered for this study, the 10 retrospectively selected patients (5 males and 5 females) were representative of typical cases in our clinic (2 Gy per single fraction, usually a door-to-door time < 1.5 h), with age ranging between 25 and 75 years and height between 155 and 178 cm (see Table S2 in the Supplementary Material). As this study was performed retrospectively, a systematic comparison of time savings between manual and automatic planning for each patient was not possible. Nonetheless, we measured the time required by two operators with different experience in TMI-TMLI planning to perform the manual procedure on one patient, which took over 2 h for a senior planner in an environment with no distractions or unexpected events, as may occur in clinical routine (see Table S3 in the Supplementary Material). A future multicenter study is currently under design to further validate the proposed automatic planning procedure developed in this study.

## Conclusion

The present study showed that automating the planning of lower extremities plans with a robust treatment plan at the junction is feasible, which makes it possible to conduct fully automated strategies in clinical practice. In terms of time savings, the required planning time has been reduced by 2 days, allowing planning of TMI-TMLI to be streamlined, thus improving its efficiency and reducing the risk of human errors. Furthermore, the presented automatic procedure will promote the standardization and automation of TMI-TMLI treatments and facilitate their clinical implementation in other institutions.

## Supplementary Information

Additional information, including the manual planning procedure description, script details, patient demographics table, and manual procedure timing table.

The zip containing the script executable used in this study.
