# Supplementary material for: Automatic planning of the lower extremities for total marrow irradiation using volumetric modulated arc therapy
Source: Strahlenther Onkol. 2022 Nov 3;199(4):412–9. doi: 10.1007/s00066-022-02014-0 (PMC10033624; doi:10.1007/s00066-022-02014-0)
Supplement: Supplementary file 1 — Additional information, including the manual planning procedure description, script details, patient demographics table, and manual procedure timing table. [file 66_2022_2014_MOESM1_ESM.docx]

**Supplementary Material**

**Automatic Planning of the Lower-Extremities for Total Marrow Irradiation Using Volumetric Modulated Arc Therapy**

**Strahlentherapie und Onkologie**

**Nicola Lambri, Damiano Dei, Victor Hernandez, Isabella Castiglioni, Elena Clerici, Leonardo Crespi, Chiara De Philippis, Daniele Loiacono, Pierina Navarria, Giacomo Reggiori, Roberto Rusconi, Stefano Tomatis, Stefania Bramanti, Marta Scorsetti, Pietro Mancosu**

Corresponding Author: Pietro Mancosu, Email: pietro.mancosu@humanitas.it

ORCID: 0000-0002-0165-7931

**S1 Manual Planning**

For the upper-body plan, the fields’ widths, perpendicular to the multileaf collimator (MLC), was set to 40 cm, while the field lengths ranged from 13 to 20 cm. Each arc overlapped with the adjacent ones for at least 2 cm on each side. The collimator angle was always set to 90° (i.e., MLC along the CC direction), except for the fields positioned on the femurs for patients where the CT acquisition extended down to the knees. In such cases, the collimator angle was set to 5°/355°, which allowed a better coverage of the PTV using only one field per lower-extremity.


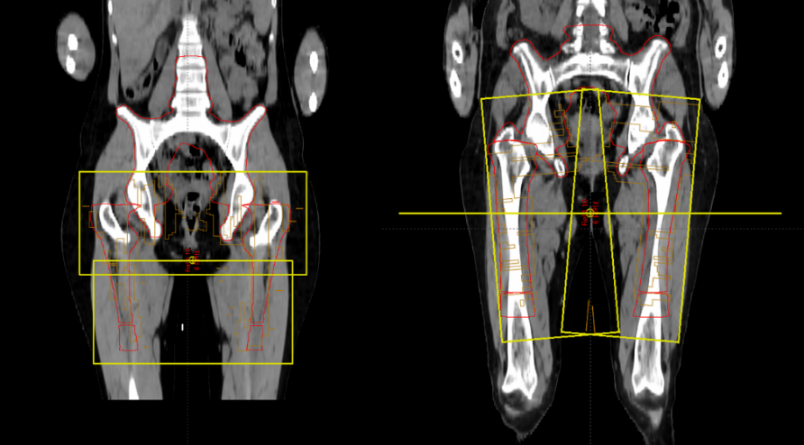


**Fig. S1** Field configuration used for the femoral region of the upper-body plan. (Left) Single isocenter and two fields with collimator angle at 90°. (Right) Single isocenter and two fields with collimator angle at 5° and 355°, with CT extending to the knees


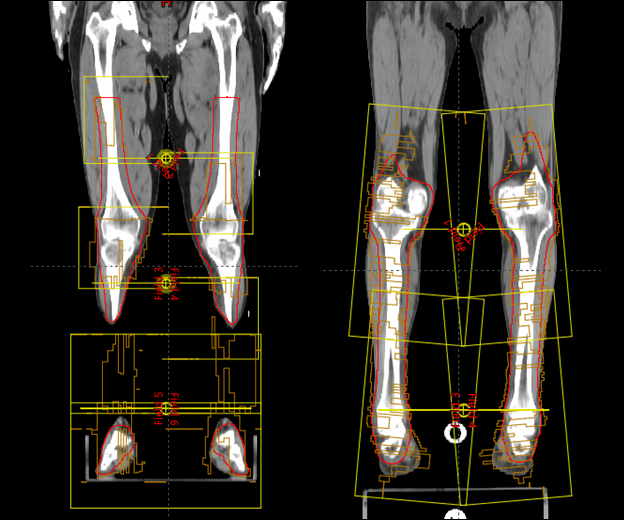


**Fig. S2** Field configurations used for the lower-extremities plan optimization. (Left) Three isocenters, six fields with collimator angle at 90°. (Right) Two isocenters, four fields with collimator angle at 5° and 355°

**S2 Script Details**

Once started, the script presents the user interface shown in Fig. S3, where the user can decide which planning step to execute (creation of junction structures, creation of control structures, and plan optimization) by selecting the corresponding checkboxes on the interface. To automatically perform the entire planning of the lower-extremities, the script needs an upper-body plan with a calculated dose, a registration of the lower-extremities CT with the upper-body CT, and a structure set containing the body and lower-extremities PTV. The user should then select from dropdown menus the following parameters:

1. Upper-body plan with a calculated dose: to create the isodose structures.
2. Lower-extremities PTV: to select the target from which the junction and control structures will be created.
3. Registration to the upper-body CT: to transform the isodose contours between the upper-body CT and lower-extremities CT frame of reference.
4. Machine name: to setup the linac model used in optimization.

The script interface allows the user to work also on the upper-body with a tab control at the top of the window. In case “Upper” is selected from the tab control, the user can create the junction and control structures for the upper-body plan, which are necessary to obtain a sigmoid dose falloff and avoid potential hotspots at the junction.


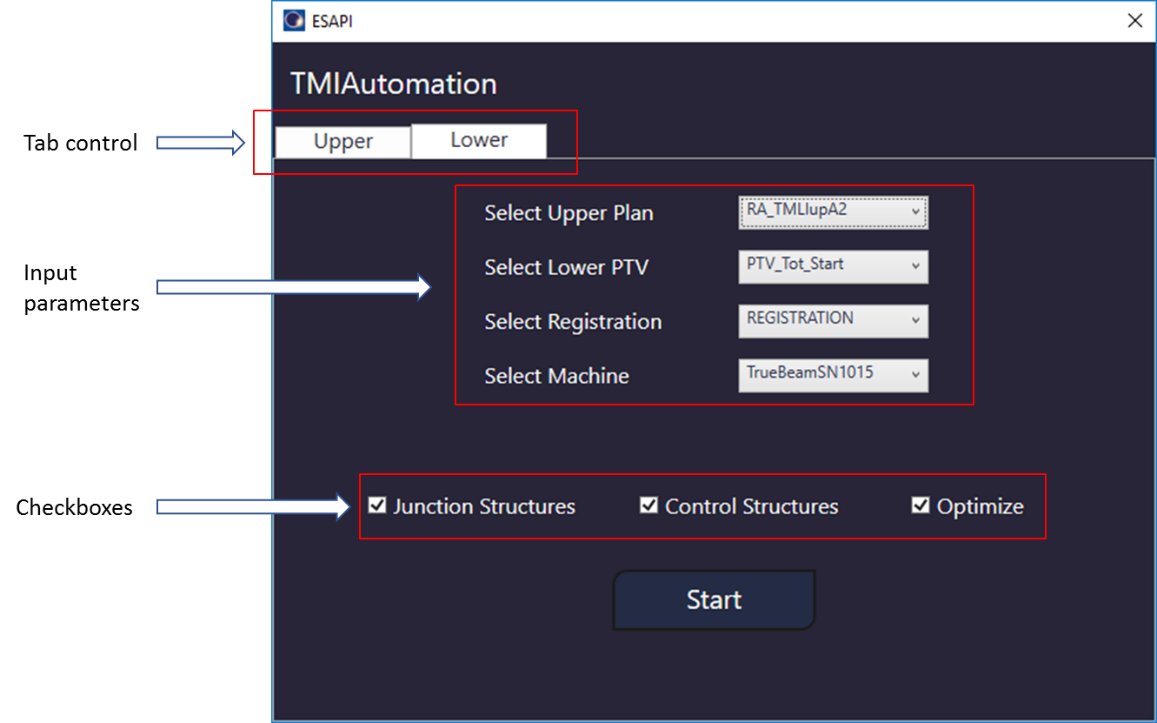


**Fig. S3** User interface of the plug-in script for the lower-extremities plan optimization

**S2.1 Lower-extremities Junction and Control Structures**

The structures PTVNoJ and PTV_J of the main manuscript are here referred to as LowerPTVNoJ and LowerPTV_J, respectively, to avoid potential confusion with the structure names of the upper-body.

For the junction structure, the script creates the 25%, 50%, 75%, and 100% isodoses (Dose_25%, Dose_50%, Dose_75%, and Dose_100%) from the upper-body plan dose distribution and propagates these structures to the lower-extremities CT using the image registration. Next, the lower-extremities PTV is cropped to the 100% isodose, while three junction substructures (PTV_J25%, PTV_J50%, and PTV_J75%) are created where the Dose_75%, Dose_50%, and Dose_25% structures intersect the lower-extremities PTV, respectively. An additional 2 cm thick substructure (PTV_J100%) is created above the PTV_J75% (toward the feet) to obtain a smoother dose gradient. The whole junction structure, LowerPTV_J, is the result of the union of the previous junction substructures. The PTV used in optimization (LowerPTVNoJ) is created by subtracting the LowerPTV_J from the lower-extremities PTV. Finally, the optimization structure REM_AUTO is added to the structure set to reduce the dose near PTV_J25%, while the Dose_100% is cropped at 3 cm below the junction (toward the head) to avoid potential hotspots in the plan sum.

The control structures consist of three structures that are needed in optimization to avoid an excess of dose to the tissues surrounding the LowerPTVNoJ. The script creates two “healthy tissue” ring structures, HT_AUTO and HT2_AUTO, using the lower-extremities PTV with an outer margin of 15 mm and inner margin of 3 mm (HT_AUTO), and an outer margin of 30 mm and inner margin of 17 mm (HT2_AUTO). Both structures are cropped from the body with a 3 mm inner margin. After that, the Body_Free_AUTO structure is generated from the body with a 3 mm inner margin and cropped with the lower-extremities PTV using a margin of 35 mm. Finally, the script removes from each control structure the contours whose area on the transversal plane is smaller than 0.5 cm^2^.

**Table S1** Summary of the main structures generated by the script for the lower-extremities plan optimization

| Structure | Purpose | Created from |
| --- | --- | --- |
| Dose_100% | Prevent hotspots in the junction region | 100% isodose level of the upper-body plan |
| LowerPTV_J | Define the lower-extremities junction: it is composed of four substructures (PTV_J25%, PTV_J50%, PTV_J75%, PTV_J100%) each one receiving a fraction of the prescribed dose | Lower-extremities PTV,  Isodose levels of the upper-body plan |
| LowerPTVNoJ | Target volume used in optimization | LowerPTV_J,  Lower-extremities PTV |
| REM_AUTO | Reduce the dose near PTV_J25% | PTV_J25% |
| HT_AUTO  HT2_AUTO  Body_Free_AUTO | Avoid an excess of dose to the healthy tissues surrounding LowerPTVNoJ | LowerPTVNoJ |

**S2.2 Upper-body Junction and Control Structures**

In case “Upper” is selected from the tab control, the user is prompted to choose the upper-body plan and upper-body PTV, already defined by the planner, to generate the junction and control structures on the upper-body CT, as shown in Fig. S4. The upper-body junction structure (UpperPTV_J) extends up to a total length of 4 cm in CC direction, starting from the most caudal slice of the selected upper-body PTV. The script splits the UpperPTV_J into four 1 cm thick substructures from bottom to top: PTV_J25%, PTV_J50%, PTV_J75%, PTV_J100%. Each of these structures is optimized to receive a fraction of the prescribed dose according to their naming convention. Finally, the script generates the optimization structure REM_AUTO used to reduce the dose near the PTV_J25%. The PTV used in optimization (UpperPTVNoJ) and the control structures (HT_AUTO, HT2_AUTO, and Body_Free_AUTO) are generated analogously as the ones of the lower-extremities plan.


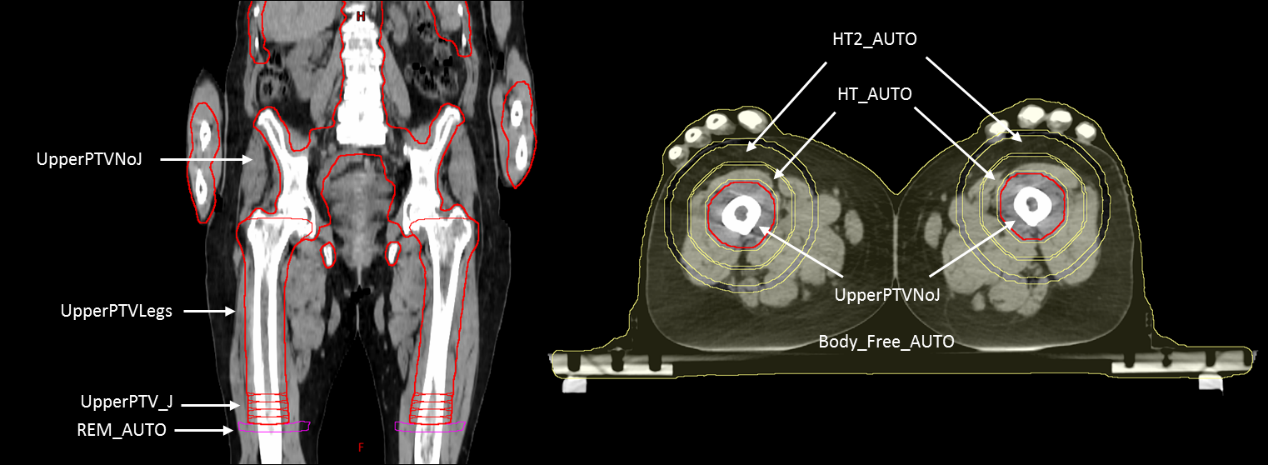


**Fig. S4** Frontal and transversal views of the main structures generated by the plug-in script on the upper-body CT (UpperPTVLegs defines the femoral PTV without the junction structure used for plan scheduling purposes)

**S2.3 Script Strengths**

We proposed an automatic solution in which the lower-extremities plan optimization can be executed without the need of human intervention. Nonetheless, the script has the flexibility to perform the three main steps (creation of junction structures, creation of control structures, and plan optimization) separately. Therefore, the planner is free to validate the structures created by the script and to correct them, if necessary, before launching the optimization. Furthermore, if desired, it is possible to run a single planning step instead of restarting the whole procedure.

**S2.4 Script Limitations**

The plug-in script has currently three limitations. The first is that the image registration between upper-body and lower-extremities CT must be performed manually, as it is not yet possible to automatically generate a new registration using ESAPI. Besides, performing an automatic registration in the femoral region, where the density variation in CC direction is small, would still require careful human validation to avoid a possible image mismatch. The second limitation is that the script typically requires 2-4 minutes to clean up the control structures by removing contours of small area as described above. Although the Contouring module of the Eclipse application allows to manually perform the same operation much faster, ESAPI does not provide a method to perform such computation directly. Nonetheless, we kept this contour removal operation in the plugin implementation to obtain a fully automatic planning procedure and avoid context switches for the planner. The third limitation is that the collimator angle for each field is always set to 90°. This configuration was preferred to keep the field geometry as simple as possible, despite an experienced planner would choose different collimator angles, as described in section 2.2. Nevertheless, the dosimetric plan quality and complexity of automatic plans showed negligible differences compared to manual plans.

**S3 Standalone Tool for Plan Optimization**

A standalone tool based on ESAPI was developed to perform the same steps of the plug-in script for the automatic isocenter and field placement, and optimization of the lower-extremities plan. Besides the configuration text files to setup the optimization objectives and calculation models, the tool needs an additional configuration file to obtain information to access the patient and the correct plan (patient ID, course ID, and plan ID), plus the machine name to setup the correct linac model. The tool was used to run overnight optimizations of the lower-extremities plan for all the 108 TMI-TMLI patients in our clinical database in order to evaluate the time required to perform batch optimizations. All plans were re-optimized in two weekends.

**S4 TMLI-TMLI Planning Workflow**


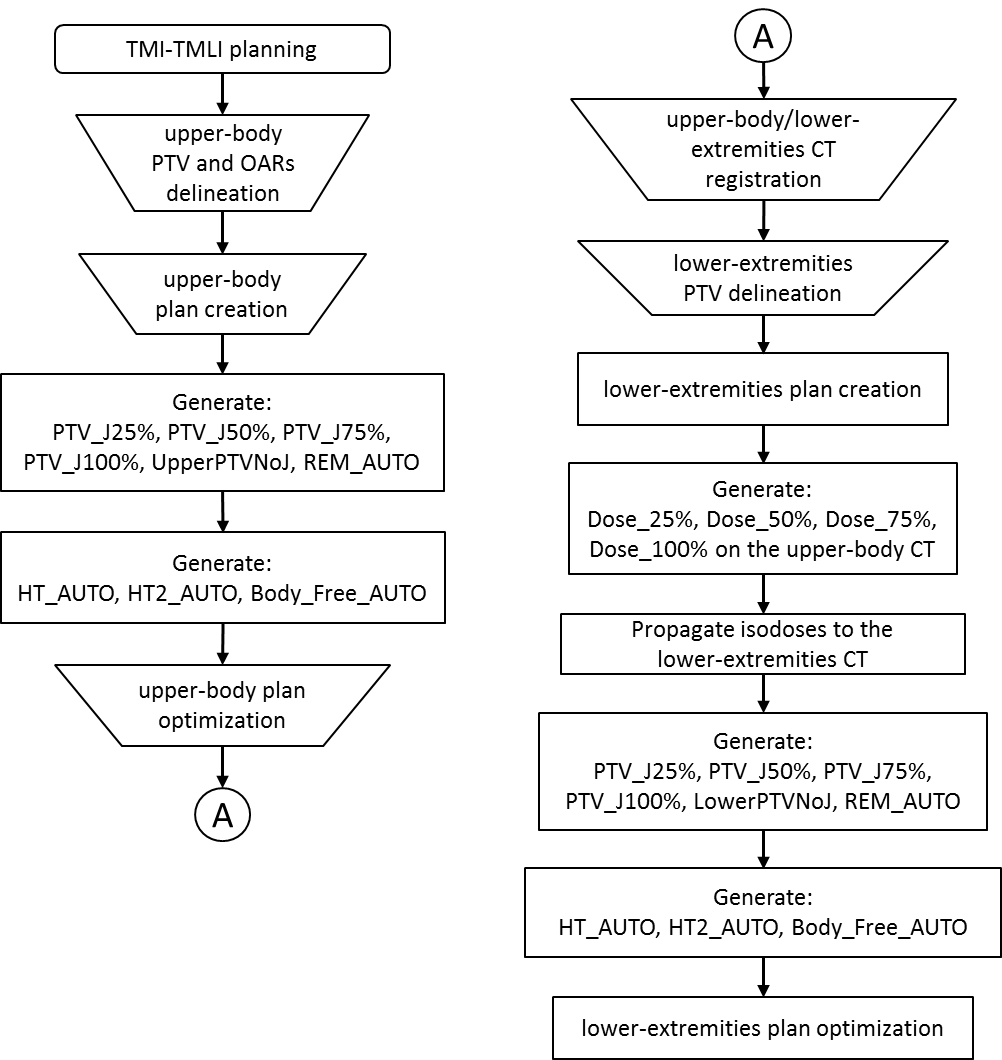


**Fig. S5** Flowchart describing the complete workflow of TMI-TMLI optimization. The steps in rectangular boxes were automated in this study

**S5 Patient Demographics**

**Table S2** Median values and range of the patient demographics considered in this study

| **Sex** | **Age (years)** | **Weight (Kg)** | **Height (cm)** | **UpperPTVNoJ Volume (cm^3^)** | **LowerPTVNoJ Volume (cm^3^)** |
| --- | --- | --- | --- | --- | --- |
| 5 males  5 females | 57  (25, 74) | 65  (48.5, 83) | 169  (155, 178) | 15 480  (11 730, 21 177) | 4 654  (3 881, 6 060) |

**S6 Dose line profiles**

**Fig. S1** Comparison of the dose line profiles along the CC direction at the junction between manual and automatic procedures, for the representative patient shown in Fig. 3 of the main manuscript. The agreement between the curves confirms the feasibility and robustness of the automatic planning procedure

**
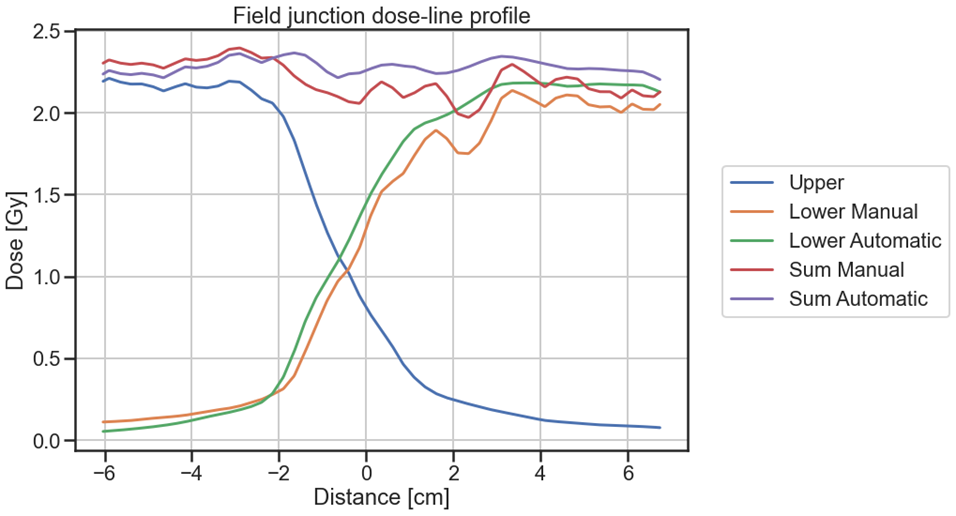
**

**S7 Manual Procedure Timing**

**Table S3** Time required by two planners to perform manually the same operations automated in this study on a single patient. Planner1 was an experienced TMI-TMLI planner

| Plan | Action | Time Planner1 (min) | Time Planner2 (min) | Time Script (min) |
| --- | --- | --- | --- | --- |
| Upper-body | Generate:  PTV_J25%, PTV_J50%, PTV_J75%, PTV_J100%, UpperPTVNoJ, REM_AUTO | 15.4 | 22.6 | 0.3 |
|  | Create:  HT_AUTO, HT2_AUTO, Body_Free_AUTO | 7.7 | 8.2 | 7.2 |
|  |  |  |  |  |
| Lower-extremities | Generate:  Dose_25%, Dose_50%, Dose_75%, Dose_100% on the upper-body CT | 1.0 | 1.1 | 0.5 |
|  | Propagate isodoses to the lower-extremities CT | 0.5 | 0.5 | 0.7 |
|  | Generate:  PTV_J25%, PTV_J50%, PTV_J75%, PTV_J100%, LowerPTVNoJ, REM_AUTO | 22.5 | 35.3 (with help of experienced planner) | 0.1 |
|  | Generate:  HT_AUTO, HT2_AUTO, Body_Free_AUTO | 7.4 | 8.5 | 3.5 |
|  | Isocenters positioning | 13.7 | 28.3 | 0 |
|  | Lower-extremities plan optimization | 75.6 | 160.8 | 55.1 |
| Total time (min) |  | 120.8 | 234.5 | 67.4 |
